# 

**Published:** 1884-05

**Authors:** 


CONTENTS.

| | PAGE. | | PAGE. |
|---|---|---|---|
| Drainage | 79 | Poetry | 90 |
| Fresh Paint | 80 | Why the Prairies are Treeless | 91 |
| Telephones and Improved Hearing | 80 | Strength of the Ancients | 91 |
| An Explanation | 81 | A Newly Discovered Hair Dye | 92 |
| The High North | 81 | Treatment of Eczema | 92 |
| Learning to Swim | 82 | Among the Lepers | 93 |
| The Sparrow and the Cat | 82 | Parasites | 95 |
| Order and Harmony | 83 | Anglo-Swiss Milk Food | 96 |
| Anxiety of a Dying Man | 83 | Life is Worth Living | 96 |
| Prevention of Pneumonia | 84 | Growth of Human Beings | 97 |
| Poisons and their Antidotes | 86 | Concentrated Oxygen | 97 |
| Insanity in France | 89 | Intellectual Sports | 98 |
| Politics and Biliousness | 89 | Medical Authorities, &c | 98 |

